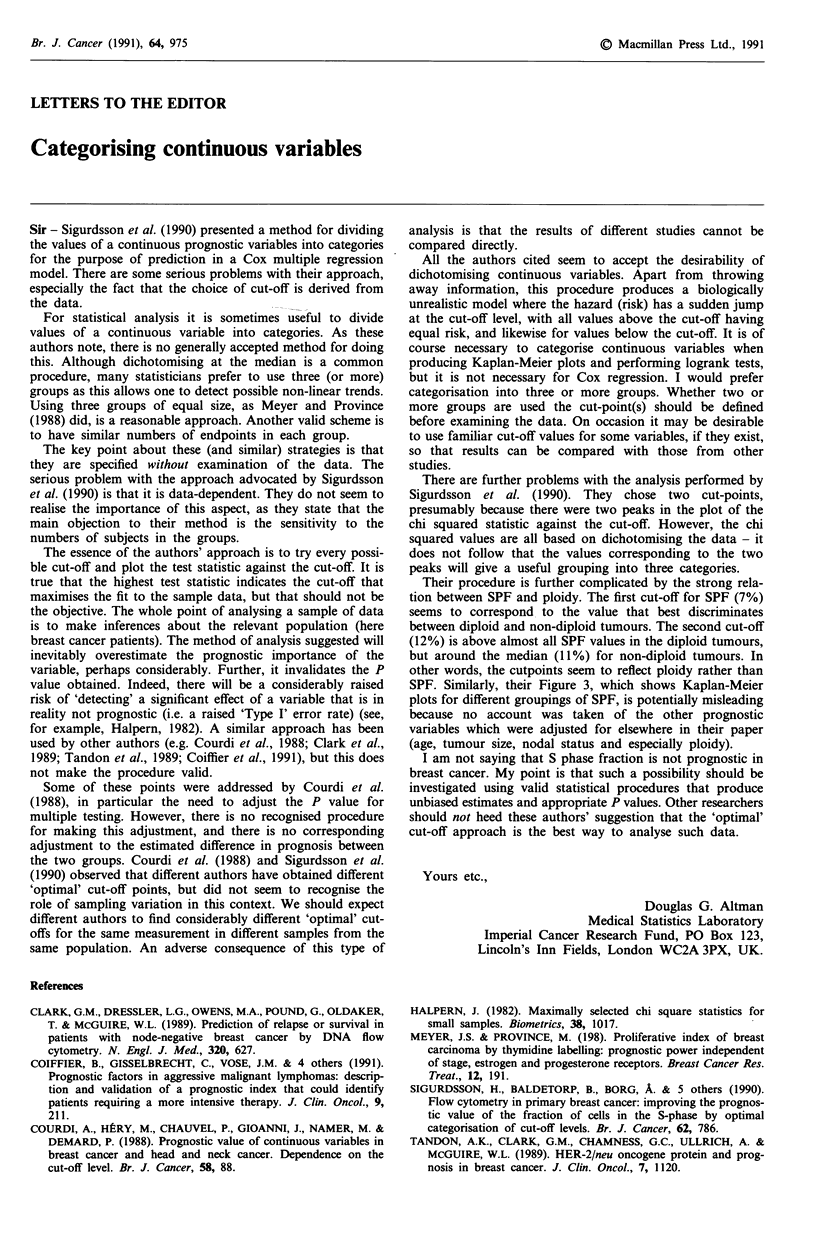# Categorising continuous variables.

**DOI:** 10.1038/bjc.1991.441

**Published:** 1991-11

**Authors:** D. G. Altman


 © Macmillan Press Ltd., 1991

### LETTERS TO THE EDITOR

### Categorising continuous variables

**Sir** – Sigurdsson *et al.* (1990) presented a method for dividing the values of a continuous prognostic variables into categories for the purpose of prediction in a Cox multiple regression model. There are some serious problems with their approach, especially the fact that the choice of cut-off is derived from the data.

For statistical analysis it is sometimes useful to divide values of a continuous variable into categories. As these authors note, there is no generally accepted method for doing this. Although dichotomising at the median is a common procedure, many statisticians prefer to use three (or more) groups as this allows one to detect possible non-linear trends. Using three groups of equal size, as Meyer and Province (1988) did, is a reasonable approach. Another valid scheme is to have similar numbers of endpoints in each group.

The key point about these (and similar) strategies is that they are specified *without* examination of the data. The serious problem with the approach advocated by Sigurdsson *et al.* (1990) is that it is data-dependent. They do not seem to realise the importance of this aspect, as they state that the main objection to their method is the sensitivity to the numbers of subjects in the groups.

The essence of the authors' approach is to try every possible cut-off and plot the test statistic against the cut-off. It is true that the highest test statistic indicates the cut-off that maximises the fit to the sample data, but that should not be the objective. The whole point of analysing a sample of data is to make inferences about the relevant population (here breast cancer patients). The method of analysis suggested will inevitably overestimate the prognostic importance of the variable, perhaps considerably. Further, it invalidates the $P$ value obtained. Indeed, there will be a considerably raised risk of 'detecting' a significant effect of a variable that is in reality not prognostic (i.e. a raised 'Type I' error rate) (see, for example, Halpern, 1982). A similar approach has been used by other authors (e.g. Courdi *et al.*, 1988; Clark *et al.*, 1989; Tandon *et al.*, 1989; Coiffier *et al.*, 1991), but this does not make the procedure valid.

Some of these points were addressed by Courdi *et al.* (1988), in particular the need to adjust the $P$ value for multiple testing. However, there is no recognised procedure for making this adjustment, and there is no corresponding adjustment to the estimated difference in prognosis between the two groups. Courdi *et al.* (1988) and Sigurdsson *et al.* (1990) observed that different authors have obtained different 'optimal' cut-off points, but did not seem to recognise the role of sampling variation in this context. We should expect different authors to find considerably different 'optimal' cut-offs for the same measurement in different samples from the same population. An adverse consequence of this type of analysis is that the results of different studies cannot be compared directly.

All the authors cited seem to accept the desirability of dichotomising continuous variables. Apart from throwing away information, this procedure produces a biologically unrealistic model where the hazard (risk) has a sudden jump at the cut-off level, with all values above the cut-off having equal risk, and likewise for values below the cut-off. It is of course necessary to categorise continuous variables when producing Kaplan-Meier plots and performing logrank tests, but it is not necessary for Cox regression. I would prefer categorisation into three or more groups. Whether two or more groups are used the cut-point(s) should be defined before examining the data. On occasion it may be desirable to use familiar cut-off values for some variables, if they exist, so that results can be compared with those from other studies.

There are further problems with the analysis performed by Sigurdsson *et al.* (1990). They chose two cut-points, presumably because there were two peaks in the plot of the chi squared statistic against the cut-off. However, the chi squared values are all based on dichotomising the data – it does not follow that the values corresponding to the two peaks will give a useful grouping into three categories.

Their procedure is further complicated by the strong relation between SPF and ploidy. The first cut-off for SPF (7%) seems to correspond to the value that best discriminates between diploid and non-diploid tumours. The second cut-off (12%) is above almost all SPF values in the diploid tumours, but around the median (11%) for non-diploid tumours. In other words, the cutpoints seem to reflect ploidy rather than SPF. Similarly, their Figure 3, which shows Kaplan-Meier plots for different groupings of SPF, is potentially misleading because no account was taken of the other prognostic variables which were adjusted for elsewhere in their paper (age, tumour size, nodal status and especially ploidy).

I am not saying that S phase fraction is not prognostic in breast cancer. My point is that such a possibility should be investigated using valid statistical procedures that produce unbiased estimates and appropriate $P$ values. Other researchers should *not* heed these authors' suggestion that the 'optimal' cut-off approach is the best way to analyse such data.

Yours etc.,

Douglas G. Altman
Medical Statistics Laboratory
Imperial Cancer Research Fund, PO Box 123,
Lincoln's Inn Fields, London WC2A 3PX, UK.

### References

CLARK, G.M., DRESSLER, L.G., OWENS, M.A., POUND, G., OLDAKER, T. & MCGUIRE, W.L. (1989). Prediction of relapse or survival in patients with node-negative breast cancer by DNA flow cytometry. *N. Engl. J. Med.*, **320**, 627.

COIFFIER, B., GISSELBRECHT, C., VOSE, J.M. & 4 others (1991). Prognostic factors in aggressive malignant lymphomas: description and validation of a prognostic index that could identify patients requiring a more intensive therapy. *J. Clin. Oncol.*, **9**, 211.

COURDI, A., HÉRY, M., CHAUVEL, P., GIOANNI, J., NAMER, M. & DEMARD, P. (1988). Prognostic value of continuous variables in breast cancer and head and neck cancer. Dependence on the cut-off level. *Br. J. Cancer*, **58**, 88.

HALPERN, J. (1982). Maximally selected chi square statistics for small samples. *Biometrics*, **38**, 1017.

MEYER, J.S. & PROVINCE, M. (198). Proliferative index of breast carcinoma by thymidine labelling: prognostic power independent of stage, estrogen and progesterone receptors. *Breast Cancer Res. Treat.*, **12**, 191.

SIGURDSSON, H., BALDETORP, B., BORG, Å. & 5 others (1990). Flow cytometry in primary breast cancer: improving the prognostic value of the fraction of cells in the S-phase by optimal categorisation of cut-off levels. *Br. J. Cancer*, **62**, 786.

TANDON, A.K., CLARK, G.M., CHAMNESS, G.C., ULLRICH, A. & MCGUIRE, W.L. (1989). HER-2/*neu* oncogene protein and prognosis in breast cancer. *J. Clin. Oncol.*, **7**, 1120.